# Impact of a Black Physician Panel Discussion on Coronavirus Disease 2019 (COVID-19) Health Education

**DOI:** 10.1017/ash.2021.2

**Published:** 2021-07-29

**Authors:** Kenisha Evans, Jannel Lee-Allen, Donald Chinemelu Okoye, Lauren Uroda, Teena Chopra, Gini Ikwuezunma, Ijeoma Nnodim Opara, Hayley Thompson

## Abstract

**Background:** Coronavirus disease 19 (COVID-19) has infected >26 million Americans with >400,000 deaths. Both Pfizer and Moderna vaccines against severe acute respiratory coronavirus 2 (SARS-CoV-2) have demonstrated 95% efficacy; yet there has been growing vaccination hesitancy, especially within communities of color. To achieve herd immunity and quell the spread of SARS-CoV-2, several strategies need to be deployed. This community-based demonstration project highlights the impact of a panel of black physicians’ ability to increase vaccination intent within a social media campaign targeted toward a black audience, namely a live question-and-answer (Q&A) event on SARS-CoV-2 vaccines. **Methods:** The social media campaign included a flyer featuring the head shots and titles of 11 black physicians. The flyer showcased a live Q&A event via Zoom video conference software. Attendees were requested to preregister with their name, e-mail address, and country of origin. **Results:** The live Q&A event was attended by 251 viewers. Geographic distribution was predominantly within the United States (~88%), but a few attendees were from the United Kingdom (~11%) and Canada (<1%), Puerto Rico (<1%), and Paraguay (<1%). One hundred twenty eight questions and comments were received from attendees. Audience questions were categorized, with predominant topics as follows: Vaccine Safety, Medical Mistrust, Vaccine Safety in Pregnancy, Vaccine Efficacy, and Vaccine Development. The top five poll results revealed: 31% of audience members were not planning to vaccinate or were not sure about vaccination, but after the event are now planning to vaccinate; 93% believed their knowledge of the C19 vaccines had increased; 95% believed it was important that the information was presented by Black health experts; 90% reported that they trusted the information presented; and 96% rated the session as “good or excellent”. **Conclusion:** Our social media project is an example of one strategy healthcare professionals can utilize to positively influence local and global communities in the mitigation of the COVID-19 pandemic. Results of this project evaluation showed that viewers responded favorably, reporting increases in vaccine acceptance and knowledge. Most respondents also affirmed the importance of having black experts involved in communicating this information. COVID-19 has disproportionately affected black communities as a result of health inequities and institutionalized racism.^1^ The event amplifies the importance of utilizing social-media–based interventions and increasing black healthcare representation to aid infection control. 1. Jones C. Why Racism, Not Race, Is a Risk Factor for Dying of COVID-19. *Scientific American* June 12, 2020.

**Funding:** No

**Disclosures:** None

